# Cure of Old Ulcers by a Recently Devised Operation

**Published:** 1871-05

**Authors:** 


Cure of Old Ulcers by a Recently Devised Operation.—Dr. Howard, of Brooklyn, presented a case of eight years' standing, which had been the result of a gun-shot wound, and was at present undergoing a rapid cure from treatment. The operation was first suggested and performed by an interne in a Paris Hospital, and for the past six or eight months had been practiced at the Charity Hospital, Blackwell's Island, by Dr. Frank H. Hamilton.

Dr. Howard said, that in the case he presented no tendency to heal occurred for years, but after implanting in it two small shreds of skin, the entire circumference of the wound showed signs of activity. In four days two other pieces were imbedded in the ulcer, where they adhered. The first portions of skin were very small and soon sloughed out; the second were larger, and instead of sloughing grew more vascular.

In this operation a piece of skin may be snipped off any part of the body, and differs from the old method—first, that there is no continuity required in the replanted portion; secondly, that one or several very minute pieces may be imbedded, according to the size of the ulcer. The only precaution that is necessary in operating being to cover the transplanted tissue with transparent adhesive plaster.—*Medical and Surgical Reporter.*

